# Hypercapnia Regulates Gene Expression and Tissue Function

**DOI:** 10.3389/fphys.2020.598122

**Published:** 2020-11-20

**Authors:** Masahiko Shigemura, Lynn C. Welch, Jacob I. Sznajder

**Affiliations:** Division of Pulmonary and Critical Care Medicine, Northwestern University, Chicago, IL, United States

**Keywords:** carbon dioxide, hypercapnia, transcriptional regulation, cellular and tissue function, lung, skeletal muscles, innate immune system

## Abstract

Carbon dioxide (CO_2_) is produced in eukaryotic cells primarily during aerobic respiration, resulting in higher CO_2_ levels in mammalian tissues than those in the atmosphere. CO_2_ like other gaseous molecules such as oxygen and nitric oxide, is sensed by cells and contributes to cellular and organismal physiology. In humans, elevation of CO_2_ levels in tissues and the bloodstream (hypercapnia) occurs during impaired alveolar gas exchange in patients with severe acute and chronic lung diseases. Advances in understanding of the biology of high CO_2_ effects reveal that the changes in CO_2_ levels are sensed in cells resulting in specific tissue responses. There is accumulating evidence on the transcriptional response to elevated CO_2_ levels that alters gene expression and activates signaling pathways with consequences for cellular and tissue functions. The nature of hypercapnia-responsive transcriptional regulation is an emerging area of research, as the responses to hypercapnia in different cell types, tissues, and species are not fully understood. Here, we review the current understanding of hypercapnia effects on gene transcription and consequent cellular and tissue functions.

## Introduction

Cells and tissues possess the ability to sense and respond to alterations in the concentration of gaseous molecules. While the understanding of how oxygen (Weir et al., 2005; Cummins et al., 2020) and nitric oxide (Haldar and Stamler, 2013) activate cellular signaling pathways to elicit adaptive responses has been well described, little attention has been given to the mechanisms by which non-excitable cells sense and respond to changes in carbon dioxide (CO_2_) levels (Shigemura et al., 2017; Cummins et al., 2020). In eukaryotic cells, CO_2_ is produced during oxidative phosphorylation and physiological CO_2_ levels in mammalian tissues (∼5%) (Shigemura et al., 2017) are significantly higher than atmospheric levels (∼0.04%)^1^ (Monastersky, 2013). In humans, severe lung disorders often impair alveolar gas exchange and lead to alveolar hypoventilation resulting in an elevation of CO_2_ levels in tissues and the bloodstream (hypercapnia) (Shigemura et al., 2017), which affects physiological consequences in the lung and other organs including brain, heart, kidney and skeletal muscles (Barnes et al., 2018). Hypercapnia has been initially proposed to be innocuous or even protective in the lung (Laffey and Kavanagh, 1999; ARDS Network, 2000) and improves outcomes in mechanically ventilated patients with acute lung injury through attenuation of stretch and sheer forces applied to the alveolar wall (volutrauma/barotrauma), cyclic recruitment-derecruitment of atelectatic areas of lung (atelectrauma), and systemic cytokine response (biotrauma) (Slutsky and Ranieri, 2013). The beneficial effects of hypercapnia have been reviewed in detail elsewhere over the past decades (Laffey and Kavanagh, 1999; Contreras et al., 2015). Contrastingly, in recent years, there is accumulating evidence that elevated CO_2_ conditions are associated with adverse pathophysiological effects on the lung (Doerr et al., 2005; Vadasz et al., 2008; Gates et al., 2013; Shigemura et al., 2018; Bharat et al., 2020) and skeletal muscles (Jaitovich et al., 2015; Korponay et al., 2020). Recent discoveries suggest that high levels of CO_2_ activate specific signaling pathways with deleterious consequences for organismal functions not only in mammals (Briva et al., 2007; O’Toole et al., 2009; Cummins et al., 2010; Wang et al., 2010; Welch et al., 2010; Vohwinkel et al., 2011; Oliver et al., 2012; Vadasz et al., 2012; Lecuona et al., 2013; Casalino-Matsuda et al., 2015; Dada et al., 2015; Selfridge et al., 2016; Kikuchi et al., 2017), but also the fly *Drosophila melanogaster* (Helenius et al., 2009; Vadasz et al., 2012), and the nematode *Caenorhabditis elegans* (Sharabi et al., 2009; Vadasz et al., 2012).

Elevated CO_2_ has also been reported to affect global gene expression in various cell types, tissues and species (Li et al., 2006; Helenius et al., 2009; Sharabi et al., 2009; Otulakowski et al., 2017; Casalino-Matsuda et al., 2018; Shigemura et al., 2018, 2019). Hypercapnia induces stress responses in animal models (Schaefer et al., 1968; Raff et al., 1983), which can alter gene expression (Zanchi et al., 2010). The increased production of stress hormones *in vivo* in response to hypercapnia was interpreted as an indirect link between elevated CO_2_ conditions and gene regulation (Taylor and Cummins, 2011). Increased ventilation stimulated by acute exposure to hypercapnia is also known to affect transcriptional regulations in respiratory muscles (Siafakas et al., 2001). More recent *in vitro* studies suggest the direct mechanisms by which hypercapnia regulates gene expression in different cell types and species (Helenius et al., 2009; Vohwinkel et al., 2011; Casalino-Matsuda et al., 2015, 2018; Shigemura et al., 2018, 2019; Korponay et al., 2020). However, a systems-level understanding of how hypercapnia effects are integrated into specific signaling pathways, and whether gene programs during hypercapnia are conserved in different cells/tissues and species still remains unclear. Here, we review recent advances in our understanding of hypercapnia-regulated gene transcription and consequent cellular/tissue functions particularly focusing on lung and skeletal muscle functions and innate immune system. We also discuss the global transcriptional response to hypercapnia and conserved alteration in transcription among the different cell types, tissues and species.

## Lung

Transcriptomic analyses have been carried out on neonatal and adult mice, as well as human bronchial epithelial cells with different CO_2_ exposure conditions (Li et al., 2006; Casalino-Matsuda et al., 2018; Shigemura et al., 2018; Bharat et al., 2020). These studies identified changes in the expression of hypercapnia-responsive genes involved in diverse cellular and tissue functions in the lung as described below.

### Lung Epithelial Function

Alveolar epithelial repair is critical for patients to recover from lung injury (Berthiaume et al., 1999). Hypercapnia, particularly hypercapnia-associated acidosis, has been proposed to improve outcomes of patients with acute lung injury, acute respiratory distress syndrome (ARDS) and ventilator-induced lung injury (VILI), which triggered the emergence of “permissive” and even “therapeutic” hypercapnia concepts (Hickling et al., 1994; ARDS Network, 2000; Contreras et al., 2015). The protective ventilation strategy was initially linked with the effects of hypercapnic acidosis on the host immune system, with the attenuation of NF-κB activity, a transcription factor that regulates inflammation, injury and repair (Contreras et al., 2015; Shigemura et al., 2017; Cummins et al., 2020). The transcriptional regulatory role of hypercapnia in anti-inflammation has been described and is discussed in detail (including deleterious effects of hypercapnia) below in section “INNATE IMMUNITY”. A transcriptomic study in a mouse model of VILI reported that hypercapnia increased the expression of α-tocopherol transfer protein, which may contribute to the protection afforded by hypercapnia in VILI (Otulakowski et al., 2017). In contrast, a recent article reported that severe hypercapnia was independently associated with higher intensive care unit (ICU) mortality in patients with ARDS (Nin et al., 2017). Delayed lung healing has been reported in patients with alveolo-pleural fistulae which manifests as a prolonged air leak from the lung surface leading to increased morbidity following surgical lung resections (Okereke et al., 2005; Singhal et al., 2010). A recent clinical study reported that intrapleural hypercapnia was associated with delayed resolution of alveolopleural fistulae in patients after thoracic surgery (Bharat et al., 2016). Interestingly, the study also suggested that the reduction of pleural CO_2_ levels was associated with faster resolution of air leaks. Alveolar epithelial cells play a role in the response to lung injury and in lung repair (Berthiaume et al., 1999; Barkauskas et al., 2013). Both proliferation and migration of alveolar epithelial cells are important for healing of lung injury (Berthiaume et al., 1999). Hypercapnia, independently of acidosis and hypoxia, has been reported to decrease cell proliferation via microRNA (miR) regulation in human alveolar epithelial cells as well as fibroblasts (Vohwinkel et al., 2011). The impaired cell proliferation by hypercapnia was associated with the miR-183-mediated transcriptional downregulation of the tricarboxylic acid cycle enzyme, isocitrate dehydrogenase-2 and consequent mitochondrial dysfunction. In a different model, hypercapnic acidosis was shown to decrease cell migration and wound repair via inhibition of NF-κB in human alveolar and bronchial epithelial cells (O’Toole et al., 2009). More recently, new evidence obtained through a network analysis of hypercapnia-responsive genes suggests that C-X-C motif chemokine 12 (CXCL12) plays a role in cell migration and wound repair in alveolar and airway epithelial cells during normoxic hypercapnia, independently of extracellular pH (Bharat et al., 2020). CXCL12 is secreted after scratch wound by lung epithelial cells (Ghosh et al., 2012) and promotes alveolar epithelial cell migration by regulating the Rac1-GTPase and cofilin activation (Kanter et al., 2015). In this study, hypercapnia decreased CXCL12 at gene expression level via reduced NF-κB activation following the Rac1-GTPase/cofilin pathway in the epithelial cells of mice and humans.

### Airway Function

Persistent hypercapnia is associated with increased disease severity and worse prognosis in obstructive lung diseases such as obesity hypoventilation syndrome (Piper, 2015) and chronic obstructive pulmonary disease (COPD) (Connors et al., 1996; Köhnlein et al., 2014; Murphy et al., 2017). More recent reports show that treating hypercapnic COPD patients with noninvasive ventilation aimed at the reduction of CO_2_ levels improved mortality (Köhnlein et al., 2014; Murphy et al., 2017) thus, supporting the notion that hypercapnia contributes to airway disease pathogenesis. We have reported that in a mouse model chronic hypercapnia increased airway smooth muscle cell contractility (Shigemura et al., 2018). Our gene network analysis suggested the “miR-133a–related RhoA/myosin light chain (MLC) phosphatase-MLC” pathway as the enriched signaling pathway of airway smooth muscle contraction, which was validated by molecular approaches with studies in mice and cell culture systems. Airway remodeling is an important part of the pathophysiology of obstructive respiratory diseases (Hirota and Martin, 2013; Prakash, 2016). It can be associated with excessive extracellular matrix deposition. In a neonatal mouse model, chronic hypercapnia was suggested to activate genes related to the composition of the extracellular matrix such as collagens and laminins (Li et al., 2006). Hypercapnia may also promote airway smooth muscle contractility altering the expression of genes involved in airway remodeling.

## Skeletal Muscles

The detrimental effects of hypercapnia are not limited to the lungs. Lung diseases affect suboptimal function of other metabolic organs such as skeletal muscles (Barreiro et al., 2015). Skeletal muscle wasting, an imbalance between protein degradation and synthesis, is frequently observed as a comorbidity in patients with chronic lung diseases such as COPD and correlates with increased morbidity and mortality (Jaitovich and Barreiro, 2018). In a murine model, normoxic hypercapnia has been shown to cause skeletal muscle atrophy via catabolic activation of the AMP-activated kinase (AMPK)/FoxO3a/E3-ubiquitin ligase muscle-specific RING finger protein-1 (MuRF1) signaling axis (Jaitovich et al., 2015). A transcriptomic study of mouse skeletal muscles revealed that normoxic hypercapnia altered the expression of genes involved in biological processes in diaphragm and soleus muscles (Shigemura et al., 2019). More recently, it has been shown to affect ribosomal biogenesis showing a marked reduction of ribosomal 45S pre-RNA in soleus and extensor digitorum longus (EDL) muscles isolated from mice exposed to hypercapnia and cultured myotubes exposed to high CO_2_ as well as quadriceps muscles from hypercapnic patients (Jaitovich et al., 2015; Korponay et al., 2020). An unbiased proteomic analysis of EDL muscles in chronic hypercapnia-exposed mice also indicated downregulation of components of “translation initiation” and “structural constituent of ribosome,” suggesting high CO_2_-mediated regulation of protein anabolism (Korponay et al., 2020). In skeletal muscle physiology, the discoveries of miRs have led to further understanding of the transcriptional complexity. MyomiRs, which represent a suite of miRs such as miR-1, miR-133, miR-208 and miR-499, are highly enriched in skeletal muscles and have distinct roles in modulating skeletal muscle proliferation and differentiation as well as the regulation of the skeletal muscle phenotype and performance (Chen et al., 2006; Williams et al., 2009; McCarthy, 2011). Several studies reported that chronic intermittent hypoxia-hypercapnia leads to slow-to-fast muscle fiber shift via either the increase in the expression of miR-1 and miR-133a (Pan et al., 2016) or the decrease in miR-208b and miR-499 (Huang et al., 2016) modulating the expression of their target transcription factors, which results in significant reduced running capacity (Pan et al., 2016).

## Innate Immunity

Hypercapnic patients with pulmonary infections have higher ICU admission and mortality (Afessa et al., 1995; Sin et al., 2005; Laserna et al., 2012), suggesting the role of elevated CO_2_ *per se* for immune system function. In studies of experimental organism model, normoxic hypercapnia resulted in altered expression of innate immune genes in *C. elegans* (Sharabi et al., 2009) and *D. melanogaster* (Helenius et al., 2009), and hypoxia and hypercapnia in *Callosobruchus chinensis* (Cui et al., 2019). In flies, hypercapnia suppressed expression of specific antimicrobial peptide genes (ex. diptericin) regulated by Relish which is an ortholog of the mammalian NF-κB, and significantly increased the mortality of the flies inoculated with bacteria (Helenius et al., 2009). A genome-wide RNA interference screen in *Drosophila* S2 cells reported 16 genes including the zinc finger homeodomain *zfh2* with human orthologs whose knockdown restored the suppression of diptericin during hypercapnia (Helenius et al., 2016). Interfering with *zfh2* in the flies significantly improved survival from *Staphylococcus aureus* infection, suggesting *zfh2* as a critical regulator of immune suppression by hypercapnia. The transcriptomic studies of hypercapnia in mice and humans revealed that normoxic hypercapnia altered the expression of multiple components of the innate immune system (Li et al., 2006; Casalino-Matsuda et al., 2018). Chronic hypercapnia suppressed the expression of inflammatory mediator genes such as interleukins, tumor necrosis factor (TNF) and chemokines (e.g., *Cxcl14*) in murine neonatal lung (Li et al., 2006). In human bronchial epithelial cells, hypercapnic acidosis also downregulated the gene expression of interleukin six (IL-6) receptor and chemokines (e.g., *CXCL14*) (Casalino-Matsuda et al., 2018). CXCL14, C-X-C motif chemokine ligand 14, is conserved between species and possesses chemoattractive activity for activated macrophages and natural killer cells (Hara and Tanegashima, 2012). In immune cells, high CO_2_ conditions selectively inhibit the gene expression of IL-6 and TNF, and decreases phagocytosis in macrophages including mouse and human alveolar macrophages (Wang et al., 2010). Furthermore, it attenuates Beclin 1 activity by increased expression of anti-apoptotic genes, BCL2 and Bcl-xL, in human macrophages, and inhibits autophagy and macrophage killing of bacteria (Casalino-Matsuda et al., 2015). Hypercapnia was reported to promote anti-inflammatory and immunosuppressive functions via activation of the non-canonical NF-κB component IKKα/RelB/p100 while inhibiting the canonical NF-κB pathway that activates host defense genes (Cummins et al., 2010; Oliver et al., 2012; Keogh et al., 2017). The hypercapnia-mediated immune suppression (impaired regulation of cytokine genes, phagocytosis and autophagy, and NF-κB signaling) occurred independently of hypoxia and pH changes. A transcriptomic study of peripheral blood in elite divers reported alterations of leukocyte gene expression profiles in response to freediving (Eftedal et al., 2016) which can cause acute hypoxia and hypercapnia (Overgaard et al., 2006). Interestingly, deconvolution of transcriptomes indicated a temporary decrease of CD8^+^ T cells and resting natural killer cells in response to acute hypoxia and hypercapnia. Furthermore, biological pathway analysis showed downregulation of genes coding for components of granule-mediated lymphocyte cytotoxicity.

## Global Transcriptional Response to Hypercapnia

**FIGURE 1:**
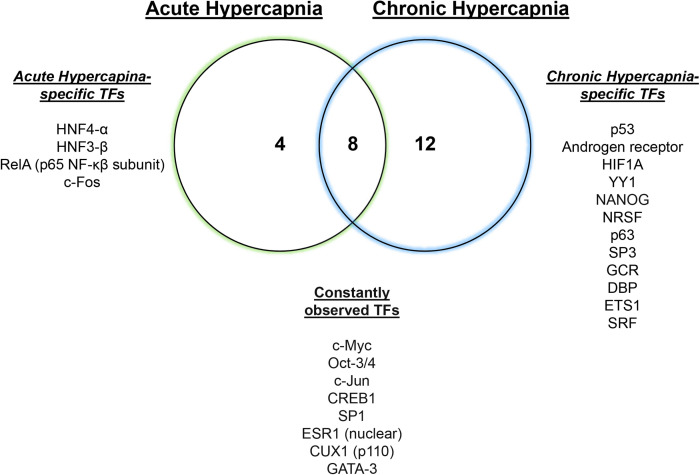
Hypercapnia-responsive transcription factors conserved in mouse tissues. Twelve or twenty hypercapnia-responsive transcription factors (TFs) were observed during acute or chronic hypercapnia conditions in mouse lung, diaphragm and soleus, respectively. Eight conserved TFs consistently inferred gene expression signatures in the tissues during hypercapnia. Modified from Shigemura et al. (2019).

We have recently reported a comparative transcriptomic study of hypercapnia (Shigemura et al., 2019) to investigate the interaction/integration/conservation of genes combining multi-tissue microarray analysis in mice with secondary analysis of available datasets in human bronchial epithelial cells (Casalino-Matsuda et al., 2018), *D. melanogaster* (Helenius et al., 2009) and *C. elegans* (Sharabi et al., 2009). We found that normoxic hypercapnia transiently increased particularly Wnt ligand and Frizzled genes in lungs and skeletal muscles of mice and in several cell lines of different tissue origin, and activated Wnt pathway homologs, which was also observed in the human bronchial cells, flies and nematodes, suggesting an evolutionarily conserved role of elevated CO_2_ in regulating Wnt signaling pathways. The Wnt signal plays a critical role in diverse biological processes and is part of a highly conserved pathway in animals (Abiola et al., 2009; Sethi and Vidal-Puig, 2010). The Wnts activate at least two distinct intracellular pathways, canonical Wnt/β-catenin or non-canonical β-catenin-independent (Sethi and Vidal-Puig, 2010). The canonical Wnt signal activates β-catenin-responsive target genes via cytosolic and nuclear β-catenin accumulation. The non-canonical signals are characterized as the calcium/calmodulin-dependent kinase II (CaMKII)-mediated Ca^2+^ signaling pathway and planar cell polarity pathway via the activation of small GTPase RhoA and Jun N-terminal kinase (JNK). The Wnts are also suggested to activate the metabolic sensor AMPK in myotubes (Abiola et al., 2009) and MuRF-1 in muscle atrophy (Rajasekaran et al., 2017). Intriguingly, we have reported that CaMKII (Vadasz et al., 2008), RhoA (Shigemura et al., 2018), JNK (Vadasz et al., 2012), AMPK (Vadasz et al., 2008; Welch et al., 2010; Jaitovich et al., 2015; Bharat et al., 2020) and MuRF1 (Jaitovich et al., 2015) are responsive to hypercapnia in the above-mentioned pathophysiological contexts. In the large-scale transcriptomic study, we inferred several potentially regulatory transcription factors for the hypercapnia-responsive genes, which are conserved amongst lung and skeletal muscle tissues (Figure 1). Some of the transcription factors, for example c-Myc (Dong et al., 2017; Zhao et al., 2017) and c-Jun (Zhao et al., 2017), are the target genes of the Wnt signaling. Transcriptional regulation of Wnt pathway genes might be of critical importance in the systems-level understanding of hypercapnia effects in organisms.

## Conclusion

**FIGURE 2:**
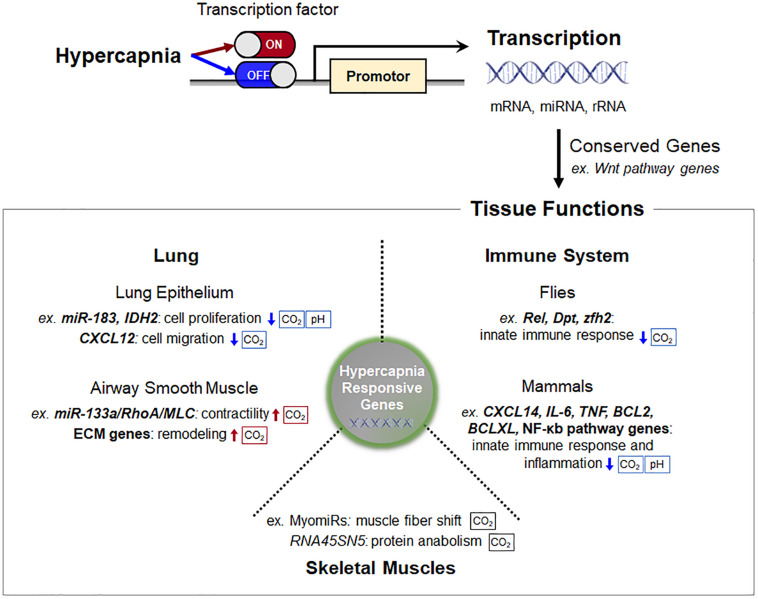
Schematic of transcription regulation and consequent tissue functions in hypercapnia. Cells, tissues, and organisms possess a complex transcriptional program that selectively promotes certain genes while simultaneously attenuating translational activity in response to elevated CO_2_ levels. Some of the hypercapnia-responsive genes, particularly Wnt pathway genes, are evolutionarily conserved in different cell types, tissues, and species. The sensing pathways that are pH-dependent are indicated. Abbreviations: IDH2, isocitrate dehydrogenase-2; CXCL12, C-X-C motif chemokine 12; MLC, myosin light chain; ECM, extracellular matrix; RNA45SN45, ribosomal 45S pre-RNA; Rel, Relish; Dpt, diptericin; zfh2, Zn finger homeodomain 2; TNF, tumor necrosis factor.

Cells, tissues and organisms possess a complex transcriptional program that selectively promotes certain genes while simultaneously attenuating translational activity in response to elevated CO_2_ levels (Figure 2). It has become evident that some of the hypercapnia-responsive genes, particularly involved in innate immune system and Wnt pathways, is evolutionarily conserved in different cell types, tissues and species. The changes in gene expression during hypercapnia appear to be mostly maladaptive in the lung, skeletal muscle and innate immune functions, which likely underlies the negative effects of elevated CO_2_ in patients with lung diseases. Our understanding of the transcriptional regulatory role of hypercapnia in organisms is still limited, but much research is being conducted in order to identify molecular CO_2_ sensing and downstream effects. Additional research is warranted to identify transcriptional regulators and how these regulators interact in physiological and pathophysiological contexts in hypercapnia.

## Author Contributions

MS, LW, and JS conceived, designed the review and wrote the manuscript. All authors read and approved the manuscript.

## Conflict of Interest

The authors declare that the research was conducted in the absence of any commercial or financial relationships that could be construed as a potential conflict of interest. The reviewer CT declared a past co-authorship with one of the authors, JS, to the handling editor.

## Footnotes

1 https://scripps.ucsd.edu/programs/keelingcurve/
